# Self-perceived burden and associated factors in Chinese adult epilepsy patients: A cross-sectional study

**DOI:** 10.3389/fneur.2022.994664

**Published:** 2022-09-13

**Authors:** Binmi Tang, Yaqian Fu, Birong Liu, Qifeng Yi

**Affiliations:** ^1^Xiangya Nursing School, Central South University, Changsha, China; ^2^Department of Nursing, Third Xiangya Hospital, Central South University, Changsha, China

**Keywords:** epilepsy, adult, Self-perceived burden, stigma, quality of life

## Abstract

**Objectives:**

Epilepsy requires long-term or lifelong treatment, and patients are prone to financial, emotional and psychological burdens that can cause psychological changes during the treatment process. This study aimed to describe the prevalence and associated factors of Self-Perceived Burden (SPB) in Chinese adult epilepsy patients, informing the treatment and nursing of epilepsy.

**Methods:**

A total of 143 adult epilepsy patients were included in this study, and the clinical questionnaire survey was conducted at the Third Xiangya Hospital of Central South University in Hunan, China, from March 2022 to June 2022. The sociodemographic and clinical characteristics of adult epilepsy patients were collected using a self-developed questionnaire, and the data on SPB, stigma and quality of life were collected through the Self-Perceived Burden Scale (SPBS), Kilifi Stigma Scale for Epilepsy (KSSE) and Quality of Life in Epilepsy Inventory-31 (QOLIE-31). Multiple linear regression analysis was used to determine the associated factors influencing SPB in adult epilepsy patients.

**Results:**

The average score of SPBS for the 143 adult epilepsy patients was 30.77 (SD = 9.06), and 89.5% of them had obvious SPB. The results of the univariate analysis showed that residence, monthly household income, duration of epilepsy, type of medication and electroencephalogram finding were associated with SPB (*P* < 0.05). In Spearman correlation analysis indicated that SPBS score were positively correlated with KSSE score (*r* = 0.510, *P* < 0.05) while negatively correlated with QOLIE-31 score (*r* = −0.506, *P* < 0.05). Multiple linear regression analysis revealed that factors such as KSSE, type of medication, residence and electroencephalogram finding accounted for 32.8% of the factors influencing SPB in adult epilepsy patients.

**Conclusion:**

89.5% of adult epilepsy patients have varying degrees of SPB, which is associated with high stigma and poor quality of life. Therefore, during the treatment and nursing of adult epilepsy patients, clinical staff should pay attention to the psychological status of patients, help them reduce their psychological burden, and guide them to develop a healthy lifestyle.

## Introduction

Epilepsy is a common chronic neurological disease that affects about 70 million people worldwide, and the total prevalence rate is 7.6%, 80% of epilepsy patients live in low and middle-income countries, such as Southeast Asia, Latin America, and sub-Saharan Africa (1, 2). There are about 10 million epilepsy patients in China, accounting for 1/7 of the world (3). Epilepsy often causes physical and psychological harm to patients, such as sudden unexpected death in epilepsy, status epileptics, falls, sleep disorder, social maladjustment, etc (4–10). These symptoms have seriously affected epilepsy patients' quality of life (11), thereby resulting in a Self-Perceived Burden (SPB). Adults are the backbone of the family and society, and adult epilepsy patients need to bear greater psychological, emotional, economic and social pressure than minors and elderly patients and are more likely to develop SPB. Therefore, it is very important to study the prevalence and associated factors of SPB in adult epilepsy patients for the treatment and nursing of epilepsy.

SPB is a common psychological characteristic in patients with chronic diseases. SPB refers to the sense of dependence, frustration and guilt of the patients on caregivers' care, and the patients are worried about the physical, emotional, psychological and economic impacts on the caregivers (12). Extensive research work has been done to understand the prevalence and associated factors of SPB. In a study of patients with chronic pain diseases, Kowal et al. (13) showed that 73% of patients with chronic pain disorders experienced a different level of SPB, and SPB is related to physical conditions and negative psychology, including depression and suicidal ideation. Adejumo et al. (14) studied patients with chronic kidney disease and found that SPB is common in patients with chronic kidney disease, which is related to the quality of life. Moreover, studies on breast cancer survivors argued that SPB mediates the relationship between self-stigma and quality of life, i.e., self-stigma can increase SPB, which in turn reduces patients' quality of life (15). However, little attention has been paid to the prevalence and associated factors of SPB in adult epilepsy patients.

In the presented study, the sociodemographic, clinical data, SPB, stigma, and quality of life of adult epilepsy patients were first investigated using a self-developed questionnaire, Self-Perceived Burden Scale (SPBS), Kilifi Stigma Scale for Epilepsy (KSSE) and Quality of Life in Epilepsy Inventory-31 (QOLIE-31). Then, the prevalence and associated factors of SPB in adult epilepsy patients were statistically analyzed, assuming that SPB is associated with high stigma and poor quality of life.

## Methods

### Study design and participants

This was a cross-sectional study conducted in the Third Xiangya Hospital of Central South University in Hunan, China, from March 2022 to June 2022. In this investigation, the inclusion criteria were patients aged 18 years or older and with a diagnosis of epilepsy that met the standard of the International League Against Epilepsy published in 2017 (16). All the investigated patients were able to read and understand normally to complete a series of questionnaires adequately, and each patient voluntarily participated in this study and signed informed consent. In addition, this study excluded patients who might affect the accuracy of the research results, including patients with intellectual and psychiatric disorders (dementia, schizophrenia, etc.), patients with other major diseases (cancer, severe liver and kidney dysfunction, etc.), patients who had used drugs that could affect the cognitive and central nervous function except for the anti-epileptic drugs (AEDs), and patients who had withdrawn midway. This study was approved by the Ethics Committee of Xiangya Nursing School of Central South University (No. E202203), and patients' privacy and legitimate rights were respected and protected. A total 159 patients were invited to participate in the study, and research staff will access the qualifications of patients. Sixteen patients were excluded for the reasons: withdrawn midway (2), severe brain disease or other serious diseases (8), unable to complete the questionnaire (6). Finally, 143 patients were included in the analysis and the effective response rate of the questionnaire was 90%, and 7 patients refused to participate in this study, accounting for 4.26% of diagnosed adult epilepsy patients.

### Data collection

Adult epilepsy patients had a face-to-face interview with a trained clinical nurse so that they were clear about the study, and then data were collected uniformly. A self-developed questionnaire was used to collect patients' sociodemographic and clinical data. Sociodemographic information includes age, gender, residence, marital status, educational level, employment status, monthly household income, and payment methods. Clinical data consisted of the duration of epilepsy, age of onset, family history of epilepsy, past medical history, etiology of epilepsy, type of medication, drug side effects, type of epilepsy, and the incidence rate in recent 3 months, electroencephalogram finding. Furthermore, patients were also asked to complete a series of questionnaires including SPBS, KEES, and QOLIE-31, which were used to assess SPB, stigma, and quality of life in adult epilepsy patients, respectively.

### Questionnaires

#### Self-perceived burden scale

The SPBS was first proposed by Cousineau et al. (12) and its Chinese version was developed by Wu and Jiang (17), and the Chinese SPBS had a high internal consistency coefficient (Cronbach'α = 0.91). In the presented study, the Chinese SPBS was used to evaluate the SPB of adult epilepsy patients, which contains three dimensions of the physical burden, emotion burden, and economic burden, with a total of 10 items. Each item is rated by a 5-point Likert-type scale, with 1–5 representing none of the time, occasionally, sometimes, often, all of the time, and the SPBS score ranges from 10 to 50. According to the SPBS score, the SPB of adult epilepsy patients is divided into four levels, i.e., none, mild, moderate and severe, corresponding to SPBS score <20, 20 ≤ SPBS score <30, 30 ≤ SPBS score <40, and SPBS score ≥40.

#### Kilifi stigma scale for epilepsy

The KSSE was respectively proposed and translated by Mbuba et al. (18) and Song et al. (19), and the Cronbach'α of the Chinese KSSE is 0.889. The Chinese KSSE was applied to assess the stigma of adult epilepsy patients, including 15 items and each item with a score of 0 to 2. The scale is rated by a 3-point Likert-type scale, and 1–3 represents not at all, sometimes, and always, respectively. The total score ranges from 0 to 30, which was divided into three levels, i.e., mild, moderate, and severe when the KSSE scores are located within 0−10, 11−20, and 20−30.

#### Quality of life in epilepsy inventory-31

The QOLIE-31was developed by Cramer et al. (20) and the Chinese version was translated by Hu et al. (21), and the Cronbach'α of the Chinese QOLIE-31 is 0.912. In this study, the Chinese QOLIE-31 was employed to estimate the quality of life of adult epilepsy patients, which includes 31 items and 7 subscales, i.e., seizure worry, overall quality of life, emotional wellbeing, energy/fatigue, cognitive functioning, antiepileptic medication effects, and social functioning. The total score ranges from 0 to 100, with a higher score indicating better quality of life.

### Statistics analysis

The collected data were analyzed using SPSS 22.0 software (SPSS, Inc, Chicago, IL, USA), and the Kolmogorov-Smirnov test was conducted to examine the distribution of SPBS score, KSSE score, and QOLIE-31 score, which found that these data were normally distributed. In the presented work, the continuous variables, including age, SPBS score, KSSE score, and QOLIE-31 score, were shown as mean ± standard deviation (SD). Categorical variables, including gender, residence, employment status, education level, etc., were described as numbers (percentages). Comparisons of SPBS scores with sociodemographic and clinical variables in adult epilepsy patients were analyzed using independent *t*-tests or one-way analysis of variance (ANOVA). Spearman correlation analysis was used to study the correlation of SPBS score with KSSE score and QOLIE-31 score, respectively. Variables with *P* < 0.05 in the independent *t*-tests or one-way ANOVA were included in the multiple linear regression analysis and *P* < 0.05 (two-sided test) was considered statistical significance.

## Results

### Participants and characteristics

Based on the inclusion criteria, a total of 143 adult epilepsy patients were included in the study. All patients' sociodemographic and clinical characteristics have been presented in Table 1. The mean age of adult epilepsy patients was 31.82 years (SD: 12.10). Seventy-two (50.3%) of them were female, slightly more than male patients, and 75 (52.4%) patients were unmarried. Eighty patients (55.9%) live in cities, higher than those in rural areas. Only 44 patients (30.8%) had a university or above, and nearly 70% of the patients have received a senior high school or below. Sixty-two patients (43.2%) were unemployed, only 51 patients (35.7%) had a monthly household income of more than 5,000 yuan, and 116 patients (81.1%) were out-of-pocket. The duration of epilepsy in 88 patients (58.1%) was more than 5 years, and approximately 90% of patients had no family history of epilepsy and past medical history. Seventy-eight patients (54.4%) were polypharmacy, and 112 patients (78.3%) worried about drug side effects. Eighty-five patients (59.4%) had more than one onset in the recent 3 months, and 74 patients (51.7%) had a abnormal electroencephalogram finding.

**Table 1:** Sociodemographics data, clinical characteristics, and SPBS score of adult epilepsy patients (*N* = 143).

**Variable**	**Number (%) or mean ±SD**	**SPBS**		
		**Mean ±SD**	***T* or *F***	***P*-Value**
**Age (years)**	31.82 ± 12.10		0.254	0.776
18–29	72 (50.3)	30.94 ± 10.00		
30–50	56 (39.2)	30.21 ± 8.17		
>50	15 (10.5)	32.00 ± 7.86		
**Gender**			1.508	0.134
Male	71 (49.7)	31.92 ± 9.46		
Female	72 (50.3)	29.64 ± 8.57		
**Residence**			3.359	0.001**
Rural	63 (44.1)	33.54 ± 8.11		
Urban	80 (55.9)	28.59 ± 9.23		
**Marital status**			1.962	0.144
Married	63 (44.1)	29.10 ± 8.05		
Unmarried	75 (52.4)	32.13 ± 9.86		
Divorced	5 (3.5)	31.40 ± 5.81		
Widowed	0 (0)	-		
**Deduction level**			2.812	0.063
Junior high school or below	46 (32.2)	33.00 ± 7.41		
Senior high school or technical secondary school	53 (37.1)	30.75 ± 9.05		
University or above	44 (30.8)	28.45 ± 10.21		
**Employment status**			1.991	0.118
Employed	57 (39.9)	28.79 ± 9.43		
Unemployed	62(43.4)	32.26 ± 8.42		
Retired	5 (3.5)	27.60 ± 6.40		
Student	19 (13.3)	32.68 ± 9.78		
**Monthly household income (yuan)**			7.191	0.001**
0–3,000	52 (36.4)	34.13 ± 8.66		
3,000–5,000	40 (28.0)	30.35 ± 8.15		
>5,000	51 (35.7)	27.67 ± 9.14		
**Payment method**			0.617	0.538
Insurance	27 (18.9)	31.74 ± 6.04		
Out-of-pocket	116 (81.1)	30.54 ± 9.64		
**Epilepsy duration (years)**			3.886	0.023*
0–5	60 (42.0)	28.35 ± 7.95		
5–10	33 (23.1)	32.12 ± 10.83		
>10	50 (35.0)	32.78 ± 8.54		
**Age of onset (years)**			1.613	0.109
<18	59 (41.3)	32.22 ± 9.21		
≥18	84 (58.7)	29.75 ± 8.88		
**Family history of epilepsy**			0.239	0.811
Yes	7 (4.9)	31.75 ± 8.26		
No	136 (95.1)	30.74 ± 9.73		
**Past medical history**			−0.756	0.451
Yes	9 (6.3)	28.56 ± 5.72		
No	134 (93.7)	30.92 ± 9.26		
**Epilepsy etiology**			0.597	0.703
Brain dysplasia	11 (7.7)	33.55 ± 5.75		
Brain trauma	24 (16.8)	29.67 ± 10.70		
Hypoxic-ischemic encephalopathy	7 (4.9)	30.29 ± 8.81		
Intracranial tumor	3 (3.5)	31.60 ± 11.56		
Infections of the central nervous system	7 (4.9)	34.86 ± 10.96		
Unknown etiology	89 (62.2)	30.39 ± 8.75		
**Type of medication**			−4.638	0.000***
Monotherapy	78 (54.4)	27.77 ± 8.88		
Polypharmacy	65 (45.5)	34.47 ± 7.95		
**Worry about drug side effects**			0.891	0.374
Yes	112 (78.3)	31.13 ± 9.18		
No	31 (21.7)	29.48 ± 8.67		
**Epilepsy type**			1.680	0.174
Focal	60 (42.0)	28.97 ± 9.72		
Generalized	28 (19.6)	32.29 ± 7.64		
Generalized and focal	8 (5.6)	32.50 ± 8.44		
Unclassified	47 (32.9)	31.28 ± 8.86		
**Seizure frequency in recent 3 months**			−0.143	0.887
<1/month	58 (40.6)	30.64 ± 9.92		
≥1/month	85 (59.4)	30.86 ± 8.49		
**Electroencephalogram finding**			−1.998	0.048*
Normal	69 (48.3)	29.22 ± 9.72		
Abnormal	74 (51.7)	32.22 ± 8.21		
**SPB score**	30.77 ± 9.06			
20 < SPB	15 (10.5)			
20 ≤ SPB <30	44 (30.8)			
30 ≤ SPB <40	63 (44.1)			
SPB≥40	21 (14.7)			

SPB, Self-Perceived Burden; *P < 0.05; **P < 0.01; ***P < 0.001.

### Univariate analysis of SPB

**Figure 1:**
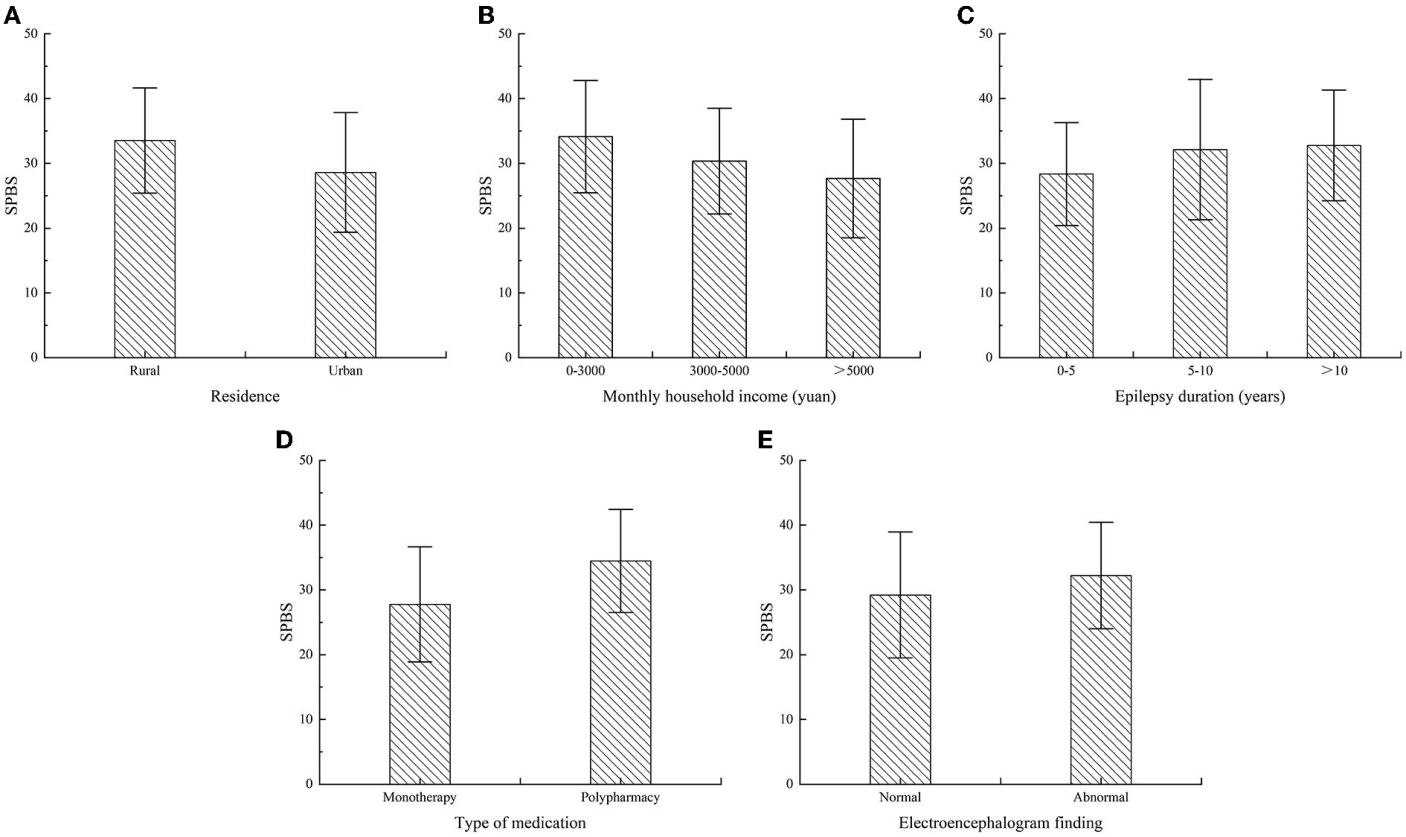
Error bar graph of SPBS score in each group in each group (N=143).

The results showed that rural patients were a higher SPBS score than urban patients (*P* = 0.001), and patients with a high monthly household income had lower SPBS score than patients with low monthly household income (*P* = 0.001), as shown in Figures 1A,B. Patients with a long duration of epilepsy had higher SPBS score than those with a short duration of epilepsy (*P* = 0.023), the SPBS score of patients on polypharmacy were higher than that on monotherapy (*P* = 0.000), and patients with abnormal electroencephalogram finding had higher SPBS score in contrast with the ones with a normal electroencephalogram finding (*P* = 0.048), as shown in Figures 1C–E. There were no significant differences (*P* > 0.05) in the SPBS score of patients among subgroups of age, gender, marital status, education level, employment status, payment methods, age of onset, family history of epilepsy, past medical history, epilepsy etiology, worry about drug side effects, epilepsy type, and seizure frequency in recent 3 months. Some other details have been presented in Table 1.

### SPBS score of adult epilepsy

The mean SPBS score of 143 patients is 30.77 (SD: 9.06), which indicates that adult epilepsy patients have a high level of SPB. 15 (10.5%) patients have no SPB, 44 (30.8%) have a mild level of SPB, 63 (44.1%) have a moderate level of SPB, and 21 (14.7%) have a severe level of SPB. The above data shows that most adult epilepsy patients have varying degrees of SPB, and each dimensions score of SPB is listed in Table 2. To further obtain the influencing factors of SPB, the SPB score of adult epilepsy patients induced by worrying the economic, physical and emotional burden of their caregivers were counted on the basis of SPBS. The mean score of economic, physical and emotional burden is 3.27 (SD: 1.13), 3.08 (SD: 1.12) and 3.02 (SD: 0.90), respectively, which suggests that epilepsy patients are most concerned about the economic strain on their caregivers.

**Table 2:** Each dimensions score of SPBS in adult epilepsy patients (*N* = 143).

**Dimension**	**Score range**	**Total score**	**Mean ±SD**
Physical burden	2–10	6.15 ± 2.24	3.08 ± 1.12
Emotion burden	6–30	18.10 ± 5.38	3.02 ± 0.90
Economic burden	2–10	6.53 ± 2.26	3.27 ± 1.13

SPBS, Self-Perceived Burden Scale.

### Correlation analysis of SPB with stigma and quality of life

**Figure 2:**
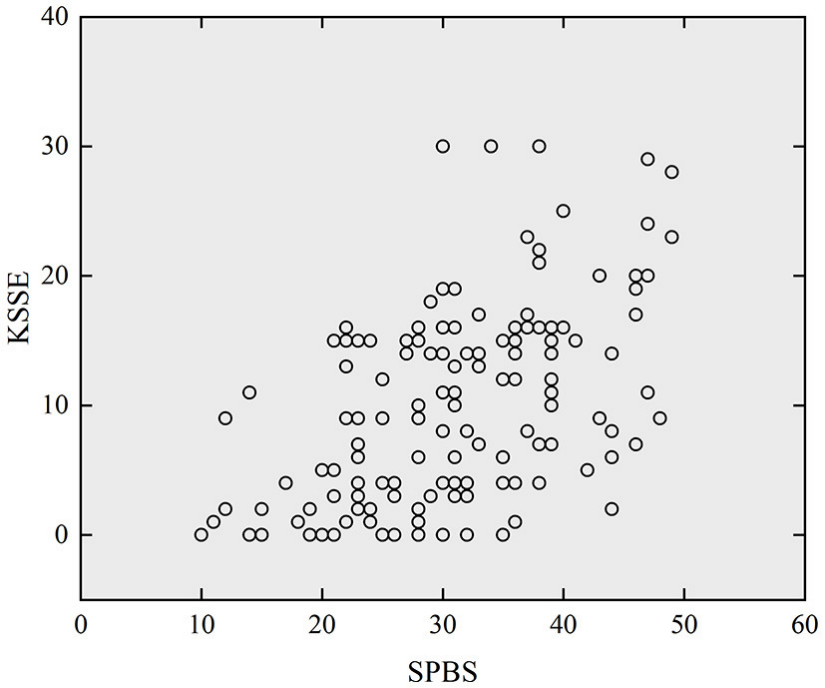
Scatterplot of SPBS versus KSSE (N=143).

**Figure 3:**
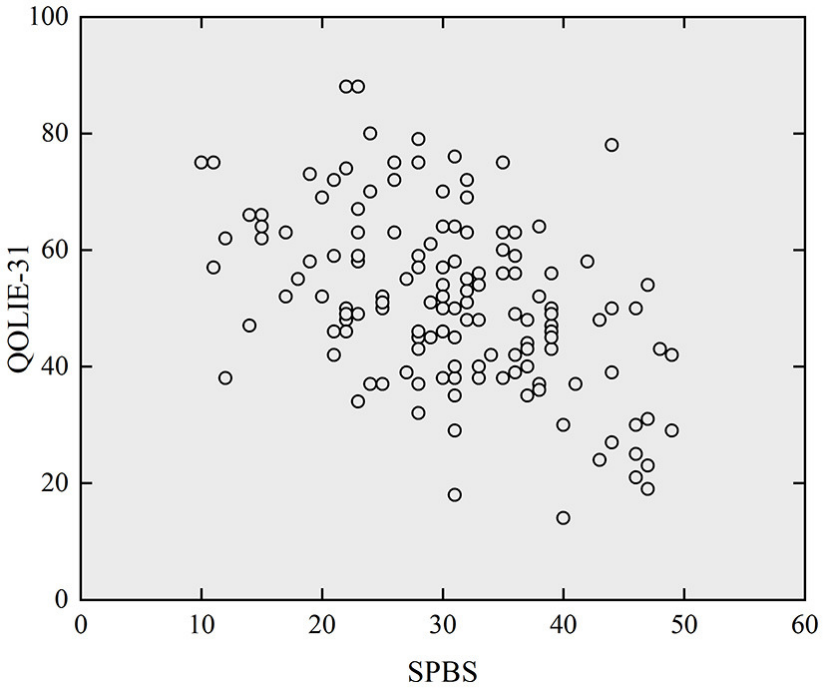
Scatterplot of SPBS versus QOLIE-31 (N=143).

Figures 2, 3 respectively presented scatterplots of SPBS vs. KSSE and QOLIE-31. It can be seen that the SPBS score of adult epilepsy patients was positively correlated with the KSSE score (*r* = 0.510, *P* < 0.05), while negatively correlated with the QOLIE-31 score (*r* = −0.506, *P* < 0.05). More details are listed in Table 3. This means that the higher the SPB, the higher the stigma, but the poorer the quality of life.

**Table 3:** Correlation analysis among SPBS score, KSSE score, and QOLIE-31 score in adult epilepsy patients (*N* = 143).

**Variables**	**Mean (SD)**	**SPBS score**	**KSSE score**
SPBS score	30.77 (9.06)	-	-
KSSE score	9.90 (7.64)	0.510**	-
QOLIE-31 score	50.82 (14.71)	−0.506**	−0.711**

SPBS, Self-Perceived Burden Scale; KSSE, Kilifi Stigma Scale for Epilepsy; QOLIE-31, Quality of Life in Epilepsy Inventory-31; SD, standard deviation. **P < 0.01.

### Multiple linear regression analysis of SPB

A multiple linear regression analysis was done using the SPBS score of adult epilepsy patients as the dependent variable and factors that were significant in the univariate analysis and Spearman correlation analysis as independent variables (*P* < 0.05). The results showed that the SPB of adult epilepsy patients was associated with KSSE, type of medication, residency, and electroencephalogram finding (*P* < 0.05), these factors account for 32.8% of the variance of SPB in adult epilepsy patients. Regression equation is *Y*_1_ = 21.21+0.477*X*_1_+3.677*X*_2_−2.878*X*_3_+2.617*X*_4_ (*Y*_1_ = SPB, *X*_1_ = KSSE, *X*_2_ = type of medication, *X*_3_ = residence *X*_4_ = electroencephalogram finding). More details are presented in Table 4.

**Table 4:** Multiple linear regression analysis for SPB and variables of adult epilepsy patients (*N* = 143).

							**Collinearity statistic**				
**Model**	**Variable**	***B***	**S.E**.	**Beta**	***t***	***P*-value**	**Tolerance**	**VIF**	**Adjusted R^2^**	***F***	***P*-value**
1	**Constant**	24.735	1.071	23.088	0.000***			0.258	50.452	0.000***
	KSSE	0.609	0.086	0.513	7.103	0.000***	1.000	1.000			
2	**Constant**	19.946	1.977		10.087	0.000***			0.294	8.139	0.005**
	KSSE	0.523	0.089	0.441	5.875	0.000***	0.884	1.131			
	Type of medication	3.881	1.360	0.214	2.853	0.005**	0.884	1.131			
3	**Constant**	24.745	2.959		8.364	0.000***			0.312	4.659	0.033*
	KSSE	0.474	0.091	0.400	5.229	0.000***	0.830	1.205			
	Type of medication	3.945	1.343	0.217	2.937	0.004***	0.884	1.131			
	Residence	−2.282	1.310	−0.155	−2.158	0.033*	0.934	1.071			
4	**Constant**	21.210	3.375		6.284	0.000***			0.328	4.390	0.038*
	KSSE	0.477	0.090	0.402	5.327	0.000***	0.829	1.206			
	Type of medication	3.677	1.333	0.203	2.758	0.007**	0.876	1.142			
	Residence	−2.878	1.295	−0.158	−2.223	0.028*	0.934	1.071			
	Electroencephalogram finding	2.617	1.249	0.145	2.095	0.038*	0.990	1.010			

KSSE, Kilifi Stigma Scale for Epilepsy; B, regression coefficient; S.E., standard error; Beta, the standardized partial regression coefficien; *P < 0.05; **P < 0.01; ***P < 0.001.

## Discussion

This study used the self-developed questionnaire, SPBS, KSSE and QOLIE-31 to conduct a cross-sectional survey of 143 adult epilepsy patients. Results showed that most adult epilepsy patients had SPB, which was influenced by the residence, monthly household income, duration of epilepsy, type of medication, and electroencephalogram finding of patients. Furthermore, SPB of adult epilepsy patients was also associated with stigma and quality of life.

SPB was prevalent in adult epilepsy patients, and the average SPBS score was 30.77 (SD: 9.06), higher than patients with chronic pain disease (22) and lower than chronic myeloid leukemia patients (23). Therefore, clinical staff need to pay more attention to the psychological status of adult epilepsy patients, develop appropriate psychological intervention measures, carry out mental health education, and prevent SPB from developing to moderate or severe (24).

Residence is an important factor in SPB in adult epilepsy patients. Rural patients had a higher level of SPB than urban patients, which is consistent with the result among urologic cancer patients (24). The following reasons may lead to discrimination against rural patients, such as the low education level of the majority of rural patients, the lack of disease awareness of the public and the characteristics of epileptic seizures, such as convulsions, and foaming at the mouth (25, 26). In addition, epilepsy patients require long-term or lifelong treatment, but rural patients have less access to medical resources and most rural patients cannot access standard anti-epileptic treatment (27). The above factors can lead to obvious SPB in rural patients. Therefore, for epilepsy patients from rural areas, clinical staff should provide appropriate assistance according to the needs of the patients to reduce psychological pressure.

The influences of monthly household income on SPB in adult epilepsy patients. Findings in this study revealed that patients with a low monthly household income had a higher SPB than patients with high monthly household income, which is consistent with several studies of SPB in breast cancer, urological cancer and cervical cancer patients (15, 24, 28). Economic income is a crucial support for epilepsy treatment, and the household with a high monthly income has a less psychological burden and a better quality of life (29). However, adult epilepsy patients have higher unemployment rates due to frequent seizures and social discrimination (30, 31). With the reduction of household income, patients cannot afford the medical costs associated with long-term treatment and may worry about the burden placed on their caregivers during treatment.

Duration of epilepsy is another associated factor on SPB in adult epilepsy patients. The findings suggested that the longer duration of epilepsy, the higher SPB in adult epilepsy patients, which is consistent with the findings of SPB in cancer patients (32). Typically, medical costs peak in the first year after diagnosis and then change depending on the severity of epilepsy, its duration, comorbidity and treatment modalities (33). The longer the duration of epilepsy, the greater the likelihood of comorbidity and the higher SPB. It has been shown that the longer the duration of epilepsy, the more depressive symptoms are experienced by epilepsy patients (34). Patients with depressive symptoms have more psychological burden and a poorer quality of life than those who without depressive symptoms (35). Therefore, clinical staff should encourage adult epilepsy patients with a long duration of epilepsy to improve their self-management ability and establish correct disease perceptions, which in turn will reduce their SPB (13).

Effects of type of medication on SPB in adult epilepsy patients. Patents on polypharmacy had a higher SPB than that on monotherapy. According to clinical statistics, more than 80% of epilepsy patients rely on AEDs treatment, and 65–70% of them can control their seizures through standard AEDs treatment (36, 37). In general, high-risk and frequent exacerbation patients need to change medication or polypharmacy, which often causes adverse effects both physical and psychological (38, 39). Moreover, patients with polypharmacy are not only prone to psychological comorbidities such as anxiety and depression (40), but also increase the risk of drug interactions and long-term drug toxicity (41). Compared to healthy people, epilepsy patients require long-term AEDs treatment in combination with other drugs, resulting in higher economic and physical burdens (42). The more types of medicines taken, the greater the physical, psychological and economic burden of the patient, and the higher the SPB.

An electroencephalogram is an important method for diagnosing epilepsy and evaluating the effectiveness of treatment (43, 44). This study showed that patients with abnormal electroencephalogram findings had a higher SPB than those with normal electroencephalogram findings. Many studies have reported that epilepsy patients with abnormal electroencephalogram finding when they stop treatment have a high risk of seizure recurrence (45–47), which suggests that patients with abnormal electroencephalogram finding need to continue anti-epileptic treatment. Clinical studies have shown that patients with epilepsy who have been seizure-free within two years but have abnormal EEG findings should also continue antiepileptic treatment to reduce the risk of recurrence (48).

Stigma is a common psychological characteristic of epilepsy patients (49). Results in this study showed that the SPB of adult epilepsy patients was positively correlated with stigma, i.e., the higher the SPB, the higher the stigma. This is consistent with the findings of SPB in breast cancer patients (15). The stigma of adult epilepsy patients was negatively correlated with quality of life, this is consistent with Tombini et al. (49) studied. Stigma can increase patients' depression, which in turn affects their quality of life (50). The stigma also affect marriage and employment for adult epilepsy patients (51). Epilepsy patients have low marriage rates due to misconceptions and stigma of the disease (52), and married adult epilepsy patients have higher family support and fewer depressive symptoms (53). In addition, stigma can accompany epilepsy patients for a long time, even after seizures have been controlled (54), and the burden of the disease can further increase the patients' stigma.

Quality of life is negatively correlated with SPB of adult epilepsy patients, i.e., the higher SPB, the poorer quality of life, which is consistent with the results of SPB in many other diseases (15, 23, 24, 55, 56). Epilepsy is a chronic neurological disease with a long treatment period. Patients with chronic diseases need to rely on their caregivers to access emotional and economic support, which can lead to SPB (13). SPB also affected patients' illness uncertainty and accordingly reduces their quality of life (23). Epilepsy patients have to seek help from their caregivers due to the effects of seizures and medication. In this case, clinical staff can provide appropriate mental health education for epilepsy patients to help them reduce their psychological burden and develop a healthy lifestyle at the same time.

### Limitations and future research directions

The presented study focused on the prevalence and associated factors of SPB in adult epilepsy patients and discussed the relationship between SPB and stigma and quality of life, which filled the gap in research on SPB in the field of epilepsy. However, there are also some limitations in this study. Firstly, all participating adult epilepsy patients were recruited from the third Xiangya Hospital of Central South University in Hunan, China, and the sample size was small and could not well-represent other cities and adult epilepsy patients in China. This is a cross-sectional study, which makes it difficult to establish a specific causal relationship between SPB and other influencing factors. In the next step, the authors plan to conduct a multi-center study to obtain a more representative sample group and conduct a longitudinal design to verify this study.

## Conclusion

This study investigated the sociodemographic and clinical data of adult epilepsy patients based on a self-developed questionnaire, SPBS, KSSE, and QOLIE-31, analyzed the SPB and its influence on adult epilepsy patients and obtained the relationship between SPB and stigma and quality of life. The results showed that 89.5% of patients had varying levels of SPB, and residence, monthly household income, duration of epilepsy, type of medication, and electroencephalogram finding were significant influencing factors of SPB in adult epilepsy patients. The SPB of adult epilepsy patients was positively correlated with stigma, while negatively correlated with quality of life.

## Data availability statement

The raw data supporting the conclusions of this article will be made available by the authors, without undue reservation.

## Ethics statement

This study was approved by the Ethics Committee of Xiangya Nursing School of Central South University (No. E202203). The patients provided written informed consent indicating their willingness to participate in this study.

## Author contributions

BT was mainly responsible for study design, data collection, analysis, and manuscript writing. YF and BL contributed to data collection. QY was mainly responsible for the analysis and interpretation of data and revision of the manuscript. All authors contributed to the article and approved the version to be submitted.

## Funding

This research was supported by the Natural Science Foundation of Hunan Province (2019JJ40469).

## Conflict of interest

The authors declare that the research was conducted in the absence of any commercial or financial relationships that could be construed as a potential conflict of interest.

## Publisher's note

All claims expressed in this article are solely those of the authors and do not necessarily represent those of their affiliated organizations, or those of the publisher, the editors and the reviewers. Any product that may be evaluated in this article, or claim that may be made by its manufacturer, is not guaranteed or endorsed by the publisher.
